# Amisulpride-induced late-onset rabbit syndrome: Case report and literature review

**DOI:** 10.1192/j.eurpsy.2022.1835

**Published:** 2022-09-01

**Authors:** I. Gundogmus, S. Tekin, A. Tasdelen Kul, Ö. Uzun, K.N. Ozmenler

**Affiliations:** 1 Kirikkale Yuksek Ihtisas Hospital, Psychiatry, Kırıkkale, Turkey; 2 Gulhane Research and Training Hospital, Psychiatry, Ankara, Turkey

**Keywords:** Antipsychotics side-effects, amisulpride, rabbit syndrome,

## Abstract

**Introduction:**

Amisulpride is an atypical antipsychotic. Rabbit syndrome(RS) may be seen after antipsychotics use a few days or long‐term application. RS occurs after more frequent typical antipsychotics and also in rare cases atypical antipsychotics.Its characterized by the involuntary rhythmic movements of the lips however involves no tongue movements.

**Objectives:**

Case report and reflection on its etiology

**Methods:**

Case report and literature review

**Results:**

A 28-year-old female with a diagnosis of schizophrenia applied with the complaints and symptoms of withdrawal, do not want to leave the house, physical anergy and avolition that started after stopped taking her medications. She was admitted to the psychiatry service and amisulpride treatment was started and was gradually increased to 800 mg/day. After 30 days of hospitalization, the patient was discharged with mild recovery. 14 days after the discharge, because of the abnormal involuntary movements in mouth, the patient applied. In clinical examination without tounge involvement, rhythmic motions were observed in the lips and jaw.Neurological examination, labrotory tests and cranial screening were all normal. She was evaluated by a private psychiatrist and was diagnosed with RS. Amisulpride treatment changed to olanzapine treatment with 15 mg/day. After two months, RS spontaneously regressed.

**Conclusions:**

The resolution of the involuntary movements following discontinuation of amisulpride in our case, supported the diagnosis of RS. Although the mechanism by which RS emerges as a side‐effect of amisulpride is not fully understood, the drug’s high affinity for and selective binding to dopaminergic D2 and D3 receptors are thought to be responsible for this involuntary motion disorder.

**Disclosure:**

No significant relationships.

